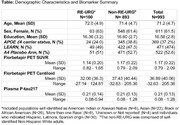# Relationship Between Plasma P‐Tau‐217 And Amyloid PET In Racial And Ethnic Underrepresented Groups (RE‐URG) Compared With Non‐RE‐URG In LEARN And A4

**DOI:** 10.1002/alz.092812

**Published:** 2025-01-09

**Authors:** Doris P. Molina‐Henry, Oliver Langford, Michael C. Donohue, Rema Raman, Roy Yaari, John R. Sims, Karen Chilcott Holdridge, Paul S. S. Aisen, Keith A Johnson, Robert A. Rissman, Reisa A Sperling, The A4 Study Team

**Affiliations:** ^1^ University of Southern California, Los Angeles, CA USA; ^2^ Alzheimer's Therapeutic Research Institute, San Diego, CA USA; ^3^ University of Southern California, San Diego, CA USA; ^4^ Alzheimer's Therapeutic Research Institute, University of Southern California, San Diego, CA USA; ^5^ Eli Lilly and Company, Indianapolis, IN USA; ^6^ Massachusetts General Hospital, Harvard Medical School, Boston, MA USA; ^7^ Gordon Center for Medical Imaging, Massachusetts General Hospital, Harvard Medical School, Boston, MA USA; ^8^ Alzheimer's Therapeutic Research Institute, Keck School of Medicine, University of Southern California, San Diego, CA USA; ^9^ Brigham and Women's Hospital and Department of Neurology, Massachusetts General Hospital, Harvard Medical School, Boston, MA USA

## Abstract

**Background:**

Individuals from diverse racial and ethnic groups are severely underrepresented in AD trials in part due to disproportionate biomarker ineligibility. Evidence from recent studies support plasma phosphorylated tau (P‐tau217) as an early marker for brain Aβ pathology and a reliable marker in predicting elevated brain amyloid PET in cognitively unimpaired adults. We examined the relationship between P‐tau217 and florbetapir PET standard uptake value ratios (SUVR) by self‐reported racial and ethnic groups in preclinical AD studies.

**Methods:**

We examined the relationship between baseline florbeptair PET SUVR and plasma P‐tau217 levels (as determined by the electrochemiluminescence immunoassay), in cognitively unimpaired adults ages 65‐85 with elevated amyloid PET (A4 Study, placebo arm, N=522) and participants who did not display elevated amyloid but were otherwise eligible for the A4 Study (LEARN, N=471). Given the small numbers across each subpopulation, participants were grouped into racial and ethnic underrepresented groups (RE‐URG; N=100) and non‐RE‐URG (N=893), based on their self‐identified race and ethnicity. A linear regression model was fit to determine differences in the relationship between plasma P‐tau217 and florbetapir PET SUVR by RE‐URG status, adjusting for age, and APOE ϵ4 carrier status.

**Results:**

Demographic characteristics of RE‐URG (N=100) and non‐URG (N=893) are displayed in the Table. Groups were balanced across, age, sex, and education, with a higher proportion of APOE ℇ4 carriers among non‐RE‐URG (38.8%) vs RE‐URG (24.0%). Mean plasma P‐tau217 levels were the same across groups (Table). Results from the linear regression model suggest that the relationship between P‐tau217 and florbetapir PET SUVR was similar across groups (p=0.22). Furthermore, APOE ℇ4 carrier status did not influence the relationship in either group (RE‐URG p=0.61; non‐RE‐URG p=0.09).

**Conclusion:**

These findings suggest that there is no difference in the relationship between plasma P‐tau217 and elevated amyloid on florbetapir PET across racial and ethnic groups. Future analyses should corroborate these findings in a larger sample size, across different RE‐URG sub‐populations and examine whether plasma P‐tau217 reflects the differential amyloid prevalence previously reported for other biomarkers of amyloid.

**Acknowledgment**: Lilly Clinical Diagnostics Laboratory for the performance of P‐tau217 testing.